# Premorbid screening of healthy students may carry latent liability for schizophrenia or bipolar affective disorder with neurocognitive and neurophenomenological methods

**DOI:** 10.1192/j.eurpsy.2022.1758

**Published:** 2022-09-01

**Authors:** I. Szendi Md Habil, A. Bagi, S. Szalóki, E. Hallgató, N. Domján, A. Kanka, B. Gál, É. Karcher, H. Pásztor, T. Jenei, O. Bóna, C. Kovács, A. Pejin, J. Daróczy, Á. Diósi, P. Pajkossy, B. Polner, G. Demeter, M. Racsmány, M. Baradits, A. Búzás, A. Dér, Z. Gingl, T. Gyimóthi

**Affiliations:** 1 University of Szeged, Department Of Psychiatry, Szeged, Hungary; 2 Kiskunhalas Semmelweis Hospital, University Teaching Hospital, Psychiatry Unit, Kiskunhalas, Hungary; 3 University of Szeged, Institute Of Psychology, Szeged, Hungary; 4 Budapest University of Technology and Economics, Department Of Cognitive Science, Budapest, Hungary; 5 Research Centre for Natural Sciences, Institute Of Cognitive Neuroscience And Psychology, Budapest, Hungary; 6 Semmelweis University, Department Of Psychiatry And Psychotherapy, Budapest, Hungary; 7 Biological Research Center, Institute Of Biophysics, Szeged, Hungary; 8 University of Szeged, Department Of Technical Informatics, Szeged, Hungary; 9 University of Szeged, Department Of Software Development, Szeged, Hungary

**Keywords:** self-agency, self-disorder, antisaccadic, actigraphy

## Abstract

**Introduction:**

This study was carried out to map psychosis spectrum disorder risk factors.

**Objectives:**

Our goal was to find what kind of instrumental methods may help to detect latent liabilities for schizophrenia and bipolar affective disorder

**Methods:**

Using online questionnaires n=710 students were screened. Groups were formed based on the inclusion criteria: N = 25 people prone to mood swings, N = 30 people prone to odd experiences and delusive thinking, and a normal control group with N = 30 people. Personality, temperament, self-experiences, affectivity scales, and cognitive screening were conducted in addition to actigraphy coupled with a mobile application for detecting subjective experiences (EMA). Furthermore, instrumental examination of self-agency, testing time interval discrimination and (re)production, eye-tracking, EEG-microstates, and laboratory testing of inflammatory, immunologic and cardio-metabolic measures of allostatic load were applied.

**Results:**

Self-experience disorders: both risk groups showed significantly higher scores than the control group (CG). Self-agency: based on incorrectly attributed responses, the positive schizotypy risk factor (PSF) group differed from the CG (p = 0.003). Antisaccade study: the PSF group showed a difference from the CG (p = 0.002). Actigraphy: based on the distributions of diurnal cumulative activities, it distinguished those with a cyclothymic risk factor (CTF) from the CG (67% probability in the k-means clustering procedure).

**Conclusions:**

Healthy students with a latent liability for schizotypy or bipolarity could be distinguished by some targeted laboratory methods. Susceptibility for bipolarity was indicated by actigraphic analyzes, and the risk for schizotypal development was indicated by deficiencies in the self-agency experience and by anti-saccadic eye movement disorders.

**Disclosure:**

No significant relationships.

